# Factor structure of the parent-rated strengths and difficulties questionnaire in a sample of Canadian children from military families

**DOI:** 10.3389/fpsyg.2023.1101407

**Published:** 2023-02-23

**Authors:** Julie Coulthard, Kerry Sudom

**Affiliations:** Department of National Defence (DND), Ottawa, ON, Canada

**Keywords:** strengths and difficulties questionnaire, scale validation, military children, exploratory factor analysis, confirmatory factor analysis, factor structure, Canadian children

## Abstract

The Strengths and Difficulties Questionnaire is a 25-item screening tool designed to measure the emotional and behavioral well-being of children. It includes five subscales including Emotional Symptoms, Conduct Problems, Peer Problems, Hyperactivity-Inattention and Prosocial Behavior. While the Strengths and Difficulties Questionnaire has been studied extensively on a global scale, it has not yet been evaluated among Canadian children from military families. This study used data collected from spouses and partners of Canadian Armed Forces members who completed a questionnaire assessing their quality of life, including the Strengths and Difficulties Questionnaire for respondents with children aged 3–16 years (*N* = 651). Using two independent randomized samples drawn from the overall group of respondents, the factorial structure was studied using exploratory factor analysis (*n* = 323) and confirmatory factor analysis (*n* = 328). Results of this study provide evidence for the factorial validity of the parent-rated Strengths and Difficulties Questionnaire for a sample of children from military families. Specifically, the exploratory factor analysis and confirmatory factor analysis supported the original proposed five-factor solution (CFI = 0.84; TLI = 0.82; SRMR = 0.073; RMSEA = 0.065) with good internal reliability of the Total Difficulties Scale and subscales. Overall, the results of this study were found to align with past research findings on the Strengths and Difficulties Questionnaire and support the future utility of this tool in assessing the well-being of Canadian children from military families.

## Introduction

Due to the occupational demands and unique nature of military life, children from military families may be at greater risk for experiencing emotional and behavioral difficulties as compared to their civilian counterparts (Chandra et al., 2010; Blamey et al., 2019; Cramm et al., 2019; Mahar et al., 2022). For example, children from these families are frequently subjected to military-induced separations, such as deployment, which disrupt the daily organization of family life and require adaptation and adjustment of all members. Studies have reported adverse effects of military separations on children, including emotional difficulties, behavioral problems, and decreased academic performance (Huebner et al., 2007; Chartrand et al., 2008; Chandra et al., 2010; Cramm et al., 2019). In particular, families may experience heightened stress, anxiety, conflict and disorientation during the pre-deployment phase as the military parent prepares to depart for an extended absence. During the deployment period itself, children from military families may suffer from reduced well-being due to changes in routine and family dynamics, lack of access to and perceived support from the military parent, as well as potential that their at-home parent may experience poorer well-being and increased stress. The post-deployment phase begins once the military parent returns home. While this is considered a happy time, the physical reintegration of the parent back into the family on a daily basis does not come without a set of challenges as it requires re-adaptation and restructuring of roles and responsibilities and can be stressful for all members (Huebner et al., 2007; Chandra et al., 2010; Coulthard, 2011; Skomorovsky and Bullock, 2017). Notably, in a study on Canadian military children, the majority of these children described deployment as one of the most stressful experiences that they have had, reporting that it negatively influenced their emotional state, physical health and academic performance (Skomorovsky and Bullock, 2017).

Research has also found that children exposed to other stressors associated with military life, such as geographic relocations and related disruptions, parental combat exposure and parental mental health conditions, display higher rates of internalizing and externalizing behaviors, along with mental disorders such as depression (Chandra et al., 2010; Blamey et al., 2019; Mahar et al., 2022). Although children with unmet psychological or emotional needs are at greater risk for developing a range of mental health issues, such issues are frequently undetected. Evidence suggests that early identification and intervention can reduce the severity and/or persistence of such difficulties (Cramm et al., 2019). It is therefore critical that the challenges that military children experience are accurately identified and measured through reliable and valid instruments that can support the development of prevention and support strategies (Hoffmann et al., 2020).

The Strengths and Difficulties Questionnaire (SDQ) is a brief screening measure that was designed to identify behavioral and emotional problems in children and adolescents. It contains four difficulties subscales assessing emotional symptoms, conduct problems, hyperactivity and inattention, and peer relationship problems, and a strengths subscale measuring prosocial behavior (Goodman, 1997; Goodman and Scott, 1999; Goodman et al., 2010). Parent and teacher versions of the SDQ are available for children aged 3–16 years of age and a self-report version was developed for youth aged 11–17 years (Hawes and Dadds, 2004; Hoffmann et al., 2020). It is a tool that is increasingly being employed in both community and clinical settings, as well as in cross-cultural research due in large part to the brevity, accessibility, and availability of it within the public domain (Mansbach-Kleinfeld et al., 2010),^1^ and it has also now been translated into over 80 languages (Español-Martín et al., 2021). The SDQ assesses both positive and negative aspects of interpersonal relationships and child and adolescent development, which also make it well suited for screening low-risk children from a population in which the majority of children are well-adjusted and psychologically healthy (Palmieri and Smith, 2007; Stone et al., 2010; Aitken et al., 2015; McAloney-Kocaman and McPherson, 2017). Further, granting the capability of employing the same instrument with multiple informants, along with the inclusion of a self-report version, increases the ability to detect psychological pathologies (Rodríguez-Hernández et al., 2012).

The factor structure of the 25-item SDQ has been extensively assessed in many different countries and across diverse cultural environments, including Australia (Hawes and Dadds, 2004; Mellor, 2005; Hayes, 2007), United States (Hill and Hughes, 2007; Ruchkin et al., 2008; He et al., 2013), United Kingdom (Goodman, 2001), Germany (Klasen et al., 2000; Downs et al., 2012), Korea (Kim et al., 2015), Finland (Koskelainen et al., 2000), Norway (Sanne et al., 2009; Brøndbo et al., 2011; Bøe et al., 2016; Lehmann et al., 2017), Sweden (Smedje et al., 1999), China (Du et al., 2008; Liu et al., 2013), Israel (Mansbach-Kleinfeld et al., 2010), and amongst Dutch (Muris et al., 2003; Stone et al., 2015), Bangladeshi (Mullick and Goodman, 2001), Spanish (Rodríguez-Hernández et al., 2012; Gómez-Beneyto et al., 2013; Español-Martín et al., 2021), Greek (Giannakopoulos et al., 2009), and Arabic children (Thabet et al., 2000). However, while some studies have also been conducted within a Canadian context (e.g., Aitken et al., 2015; Hoffmann et al., 2020), no evaluation of the SDQ based on Canadian Armed Forces military children has been conducted to date.

Many studies have confirmed the original proposed 5-factor structure, with results finding support for the model (Goodman, 1997, 2001; Smedje et al., 1999; Hawes and Dadds, 2004; Van Roy et al., 2008; Sanne et al., 2009; He et al., 2013; Niclasen et al., 2013; Kersten et al., 2016). However, mixed results concerning the factorial validity of the five-factor structure have been found through other studies using confirmatory factor analysis. Some have found a poor fit to the data using a five-factor model (Mellor and Stokes, 2007; D’Acremont and Van der Linden, 2008; He et al., 2013), while other studies have found support for alternative models representing the best fit, such as a 6-factor (Palmieri and Smith, 2007; McCrory and Layte, 2012; McAloney-Kocaman and McPherson, 2017) or a 3-factor solution (Dickey and Blumberg, 2004; Goodman et al., 2010). Looking at Canadian data, the results of a confirmatory factor analysis using a community sample of children from civilian families suggested that the five-factor model fit the data well, with evidence provided for the factorial validity and reliability of the parent-rated SDQ (Aitken et al., 2015; Hoffmann et al., 2020).

While it is not entirely unexpected in psychometric analyses to observe patterns of deviation in factor structure, it has been suggested that the contextual and cultural differences in populations of study and subsequent potential for variability in how participants may interpret the items may underlie the varying patterns observed in the factor structure of the SDQ (Goodman, 2001; Dickey and Blumberg, 2004; Stone et al., 2010; McAloney-Kocaman and McPherson, 2017). Indeed, the multicultural, multilingual, and multiple informant nature of the SDQ likely contributes to a greater complexity in the determination of the optimal factor structure (Garrido et al., 2018). Critical to ensuring that the SDQ is being applied correctly and can therefore be employed as an effective screening and outcome tool is confirming that the population-specific psychometric properties are available and can enable appropriate interpretations of the data gathered (McAloney-Kocaman and McPherson, 2017). Thus, the aim of this present study was to analyze the factor structure of the SDQ based on a sample of Canadian children from military families. First, an exploratory factor analysis was performed to identify the latent traits underlying the factors and assess the construct validity of the tool. Following the determination that the factor structure verified the pattern of factor loadings, a confirmatory factor analysis was conducted to identify the number of underlying dimensions and covariances between the factors, predicting the theoretical model proposed for the SDQ (Goodman, 2001), adopting a similar methodological approach that has been employed in past research (e.g., Hill and Hughes, 2007; Mansbach-Kleinfeld et al., 2010; Gómez-Beneyto et al., 2013; Haynes et al., 2013; Caci et al., 2015; Azzopardi et al., 2016).

## Materials and methods

### Respondents and procedure

The 2018 quality of life (QoL) survey of spouses and partners of Canadian Armed Forces (CAF) members was designed to assess the impact of the military lifestyle on the well-being of spouses and partners of CAF members. The 2018 QoL survey was administered between fall 2017 and fall 2018 to spouses and common-law partners of CAF Regular Force members. The survey was mailed to the home address based on a random stratified sample of 8,819, with responses returned from a total of 1,489 CAF spouses, yielding an adjusted response rate of 16.9%. The participants were given the option to complete the surveys in either of Canada’s official languages (i.e., English or French) and by pen-and-paper or electronically. Participants were informed that their responses would be anonymous and that the results would only be reported in aggregate (Skomorovsky and Wang, 2020). For the current study, analyses were limited to respondents who reported being the parent of at least one child who was between 3 and 16 years old, which was 43.7% of the respondents (*N* = 651). In order to cross-validate the factor models, this full sample (*n* = 651) was then divided into half with two random subsamples of roughly the same size generated for each analysis to be conducted separately (i.e., *n* = 323 for sample 1 and *n* = 328 for sample 2).

### Respondent characteristics

Demographic characteristics of this sample are presented in Table 1. The majority of respondents were women (93.4%), and 15.5% were military members. A little over one-third of respondents’ military spouses were in the Army (36.8%) and had deployed in the past 2 years (33.7%). In terms of their family characteristics, the majority of respondents (79%) indicated that they had two or more children and 15.1% of all respondents reported that they had at least one child with either a mental or physical disability.

**Table 1:** Un-weighted sample characteristics for participants (*n* = 651 with children older than 2 and less than 17 years of age).

Demographics	Category	Count	% (95% CI)
Sex^1^	Men	43	6.6 (4.9–8.7)
	Women	605	93.4 (91.3–95.1)
Age group (years)^1^	20–29	52	8.4 (6.4–10.8)
	30–39	335	54.3 (50.4–58.2)
	40–49	208	33.7 (30.1–37.5)
	50–59	22	3.6 (2.3–5.3)
	60 or older	-	-
CAF member^1^	Yes, currently in the CAF (regular force)	100	15.5 (12.9–18.5)
	Yes, former member	45	8.5 (6.4–96.6)
CAF spouse – sex^2^	Male	613	94.5 (92.5–96.0)
	Female	36	5.5 (4.0–7.5)
CAF spouse – rank^2^	Senior Officer	97	15.0 (12.4–17.9)
	Junior Officer	88	13.6 (11.1–16.4)
	Senior NCM	223	34.5 (30.9–38.2)
	Junior NCM	239	36.9 (33.3–40.7)
CAF spouse – element^2^	Sea	155	24.1 (20.9–27.5)
	Land	237	36.8 (33.1–40.6)
	Air	206	32.0 (28.5–35.7)
	Other (e.g., special forces)	46	7.2 (5.3–9.3)
CAF spouse – deployed on mission in past 2 years^2^	Yes	218	33.7 (30.2–37.5)
	No	428	66.3 (62.5–69.8)
Number of children^2^	1	137	21.0 (18.0–23.9)
	2	361	55.5 (51.6–59.1)
	3	106	16.3 (13.5–19.2)
	4	40	6.1 (4.5–8.0)
	5	7	1.1 (0.3–1.9)
At least one child with a disability (physical or mental)^2^	Yes	98	15.1 (12.5–18.0)
	No	550	84.9 (82.0–87.5)
Gender of child for SDQ^2^	Male	368	57.0 (53.1–60.7)
	Female	278	43.0 (39.3–46.9)
Age of child for SDQ (years)^2^	3–4	149	22.9 (19.8–26.2)
	5–6	111	17.1 (14.3–20.1)
	7–8	108	16.6 (13.9–19.6)
	9–10	91	14.0 (11.5–16.8)
	11–12	78	12.0 (9.7–14.6)
	13–14	65	10.0 (7.9–12.5)
	15–16	49	7.5 (5.7–9.7)

NCM, non-commissioned member. Rounding may result in percentages not equaling 100. ^1^Individual variables on spouse respondent. ^2^Household/family variables.

Respondents with more than one child were asked to complete the SDQ based on selecting the child who they were most concerned about in terms of their behaviors, adjustment, and well-being. The majority of children being reported upon were male (57.0%) and between the ages of 3–8 years (56.6%).

### Measures

The parent-rated version of the Strengths and Difficulties Questionnaire (SDQ) was used to assess behavioral and emotional difficulties among CAF children. The SDQ is a 25-item screening scale of psychosocial problems for children and consists of five subscales, each containing five items. The scale measures emotional symptoms (e.g., “*Has many worries or often seems worried*”), prosocial behavior (e.g., “*Is kind to younger children*”), peer relationship problems (e.g., *“Often fights with other youth or bullies them”*), conduct problems (e.g., *“Often loses temper”*), and hyperactivity-inattention problems (e.g., *“Is restless, overactive, cannot stay still for long*”). Each item is rated on a 3-point scale (0 = *Not True*; 1 = *Somewhat True*; and 2 = *Certainly True*) thereby yielding a subscale score for each dimension that ranges from 0 to 10. A higher score is indicative of more problems (i.e., greater difficulties) for all subscales, with the exception of the prosocial scale where a higher score corresponds to fewer difficulties in prosocial behavior and reflects strengths. The scores on the subscales (excluding prosocial behaviors) can be used to create a total difficulties score, with a range from 0 to 40 (Goodman, 1997; Stone et al., 2010).

### Statistical analyses

Descriptive statistics and the exploratory factor analysis were calculated using the Statistical Package for Social Sciences (SPSS) version 26.0, while the confirmatory factor analysis was conducted using STATA version 14. Specifically, an exploratory factor analysis using principal-axis factoring was used to explore the factor structure and assess the underlying structure for the 25 items of the SDQ (*n* = 323). Since children’s behaviors and emotional states were expected to be correlated, an oblique (Promax) rotation was used. To establish the number of factors to retain, visual examination of the scree plot and eigenvalues based on Kaiser’s criterion was conducted. Following the advice of Field (2013: 692), factor loadings less than 0.3 were suppressed. Reliability of the sample was calculated by interpreting Cronbach’s alpha coefficient (α) for each subscale. Next, in order to be able to predict how well the factor structure will fit any data using the scale, a confirmatory factor analysis was conducted on the five-factor structure identified through the exploratory factor analysis (*n* = 328). Model fit was evaluated using the following indices for consensus and convergence: the Comparative Fit Index (CFI); the Tucker-Lewis Index (TLI); the Root Mean Square Error of Approximation (RMSEA); and the Standardized Root Mean Square Residual (SRMR). Criteria used to determine goodness of fit were based on Hu and Bentler (1999) recommendations (i.e., RMSEA <0.06 for good fit, ≤0.08 for acceptable fit; CFI/TLI >0.90 for good fit, 0.8–0.9 acceptable fit; SRMR <0.08) and consistent with indicators applied in related research (Kersten et al., 2016).

## Results

In examining the proportion reporting “*Certainly True*” to “*Somewhat True*” as displayed in Table 2, the items endorsed at the highest rates were observed to be those within the Prosocial subscale, such as “*Is considerate of other people’s feelings*” (97.7%) and “*Is kind to younger children*” (96.6%). The highest rates of difficulties were noted to be items within the Emotional (“*Is nervous in new situations, loses confidence easily*” 65.3%) and within the Hyperactivity-Inattention subscales (“*Is easily distracted, concentration wanders*”; 63.1%). Conversely, the difficulties items endorsed at the lowest rates tended to be found within the Conduct Problems subscale, such as “*Steals from home, school or elsewhere*” (5.9%) and “*Often fights with other youth or bullies them*” (14.4%), with respondents generally reporting fairly overall low rates of problematic behavior manifesting in their children.

**Table 2:** Factor loadings based on principal axis factoring.

		Factor loadings (*n* = 323)				
Item: *My child….*	“Certainly True” or “Somewhat True” % (95% CI) (*n* = 651)	Factor 1: Hyperactivity-inattention	Factor 2: Prosocial behaviour	Factor 3: Peer problems	Factor 4: Emotional symptoms	Factor 5: Conduct problems
2. Is restless, overactive, cannot stay still for long (hyperactivity-inattention)	54.2 (50.3–58.0)	0.873				
10. Is constantly fidgeting or squirming (hyperactivity-inattention)	49.5 (45.6–53.3)	0.841				
15. Is easily distracted, concentration wanders (hyperactivity-inattention)	63.1 (59.4–66.8)	0.754				
25. Has good attention span, sees work through to the end (hyperactivity-inattention)	70.4 (66.7–73.9)	−0.561				
21. Thinks things out before acting (hyperactivity-inattention)	74.7 (71.2–77.9)	−0.325				
20. Often offers to help others (parents, teachers, children) (prosocial behaviour)	93.1 (90.9–94.8)		0.710			
9. Is helpful when someone is hurt, upset or feeling ill (prosocial behaviour)	95.4 (93.5–96.8)		0.693			
4. Shares readily with other youth, for example books, games food (prosocial behaviour)	93.1 (90.9–94.8)		0.676			
17. Is kind to younger children (prosocial behaviour)	96.6 (95.0–97.8)		0.644			
1. Is considerate of other people’s feelings (prosocial behaviour)	97.7 (96.3–98.6)		0.462			
14. Is generally liked by other youth (peer problems)	96.0 (94.3–97.3)			−0.684		
6. Would rather be alone than with other youth (peer problems)	39.5 (35.8–43.3)			0.598		
11. Has at least one good friend (peer problems)	90.3 (87.8–92.4)			−0.568		
19. Is picked on or bullied by other youth (peer problems)	33.3 (29.8–37.1)			0.534		
23. Gets along better with adults than with other youth (peer problems)	42.4 (38.6–46.3)			0.526		
13. Is often unhappy, depressed or tearful (emotional symptoms)	30.1 (26.7–33.7)			0.392	0.302	
24. Has many fears, is easily scared (emotional symptoms)	44.4 (40.6–48.3)				0.802	
8. Has many worries or often seems worried (emotional symptoms)	50.4 (46.6–54.3)				0.720	
16. Is nervous in new situations, easily loses confidence (emotional symptoms)	65.3 (61.6–68.9)				0.644	
3. Often complains of headaches, stomach-aches or sickness (emotional symptoms)	30.3 (26.8–33.9)				0.306	
18. Often lies or cheats (conduct problems)	29.3 (25.8–32.9)					0.743
22. Steals from home, school or elsewhere (conduct problems)	5.9 (4.3–7.9)					0.672
12. Often fights with other youth or bullies them (conduct problems)	14.4 (11.8–17.2)					0.458
5. Often loses temper (conduct problems)	61.8 (58.0–65.5)					0.426
7. Generally well-behaved, usually does what adults request (conduct problems)	93.5 (91.4–95.2)		0.392			−0.390
Cronbach’s alpha		0.81	0.74	0.68	0.76	0.67

### Exploratory factor analysis

The factorability of the 25 items in the SDQ was examined. The Kaiser-Meyer-Olkin (KMO) measure of sampling adequacy was.85, well above the commonly recommended value of.6. Bartlett’s Test of Sphericity was significant (*χ*^2^ (300) = 2403.369, *p* < 0.05), rejecting the null hypothesis of an identity matrix and providing further support that the data were sufficient to perform an exploratory factor analysis. The diagonals of the anti-image correlation matrix were also all over 0.5 and no communalities were under.2. A visual inspection of the scree plot was conducted. Given these overall indicators, factor analysis was deemed suitable for this scale.

Principal axis factoring of the SDQ initially yielded six factors with eigenvalues >1 (i.e., 6.082, 2.542, 2.307, 1.528, 1.299 and 1.008), which accounted for 46% of the total variance. However, this sixth factor was observed to account for only an additional 2% of the total variance compared to the expected 5-factor solution. Also just two items loaded into it, neither of which conceptually fit into a logical separate dimension separate from the original five proposed, nor did it align with what had been found in the literature. As such, a principal axis factoring was again performed; this time with a fixed five-factor extraction. The rotated factor solution (Table 2) in the fixed five-factor model shows that the items loading on the first factor (accounting for 24% of the variance) were suggestive of hyperactivity-inattention problems. The second factor (34% of the variance) reflected prosocial behavior. The third factor (43% of the variance) reflected emotional symptoms. The fourth factor (49% of the variance) reflected peer relationship problems and the fifth factor (55% of the variance) reflected conduct problems. Only two items cross-loaded in the rotated solution, “*My child is often unhappy, depressed or tearful*” and “*My child is* generally well-behaved, usually does what adults request.” More specifically, the first item should have loaded onto the Emotional subscale but instead loaded slightly higher on the Peer Problems subscale. Although it did not load the highest on that factor, the decision was made to retain the first cross-loading item onto the Emotional subscale in order to correspond to the subscales used in the literature and to better fit the conceptual underpinnings of this construct. Next, while the second item loaded higher, albeit only slightly, on the Prosocial subscale, the decision was made to retain it on the Conduct subscale, in accordance with the literature.

While the observed pattern of correlations suggested mutual associations across the five subscales of the SDQ (Table 3), the correlations between scale scores were small to moderate in magnitude, thereby indicating their distinctness in each of the dimensions. Correlations were positive across the four difficulties factors, while the strengths subscale, the Prosocial factor, correlated negatively with all difficulties factors but the Emotional factor.

**Table 3:** Factor correlation matrix (*n* = 323).

Factors	F1	F2	F3	F4	F5
F1. Hyperactivity-inattention	1.000				
F2. Prosocial behaviour	−0.444	1.000			
F3. Emotional symptoms	0.211	−0.417	1.000		
F4. Peer problems	0.347	−0.036	0.317	1.000	
F5. Conduct problems	0.418	−0.527	0.459	0.319	1.000

### Confirmatory factor analysis

In the confirmatory factor analysis, the model converged, and all estimates were within bounds. Model fit was evaluated with multiple indicators, with CFI and TLI considered adequate above 0.80 and good above 0.90, SRMR considered good below 0.10, and RMSEA considered adequate below 0.10 and excellent below 0.05 (Hu and Bentler, 1999; Kline, 2015). Fit indices approached all these levels, which indicated that the five-factor model had decent fit compared to the one-factor model, with RMSEA = 0.065, SRMR = 0.073, CFI = 0.84 and TLI = 0.82 (Table 4). Table 5 displays the standardized coefficients and their standard errors for each of the individual items of the SDQ.

**Table 4:** Confirmatory factor analysis for the five-factor model of the SDQ (*n* = 328).

	Criterion of fit	Index value		Fit/No fit	
		One-factor model	Five-factor model	One-factor model	Five-factor model
CFI	>0.95	0.517	0.842	No fit	No fit
TLI	>0.95	0.473	0.821	No fit	No fit
RMSEA	<0.06	0.113	0.065	No fit	No fit
SRMR	<0.08	0.102	0.073	No fit	Fit

CFI, comparative fit index; TLI, Tucker-Lewis index; RMSEA, root mean square error of approximation; SRMR, standardized root mean square residual.

**Table 5:** Confirmatory factor analysis: Standardized coefficients and standard errors for individual items (*n* = 328).

Item	Factor	Coefficient	SE
10. Is constantly fidgeting or squirming	Hyperactivity-inattention	0.762	0.034
15. Is easily distracted, concentration wanders		0.693	0.038
2. Is restless, overactive, cannot stay still for long		0.759	0.034
25. Has good attention span, sees work through to the end		−0.683	0.040
21. Thinks things out before acting		−0.489	0.051
9. Is helpful when someone is hurt, upset or feeling ill	Prosocial behaviour	0.748	0.038
20. Often offers to help others (parents, teachers, children)		0.572	0.047
17. Is kind to younger children		0.503	0.052
4. Shares readily with other youth, for example books, games food		0.532	0.050
1. Is considerate of other people’s feelings		0.673	0.042
8. Has many worries or often seems worried	Emotional symptoms	0.823	0.029
24. Has many fears, is easily scared		0.780	0.316
16. Is nervous in new situations, easily loses confidence		0.627	0.042
3. Often complains of headaches, stomach-aches or sickness		0.465	0.051
13. Is often unhappy, depressed or tearful		0.594	0.044
14. Is generally liked by other youth	Peer problems	0.657	0.052
6. Would rather be alone than with other youth		−0.497	0.057
23. Gets along better with adults than with other youth		−0.327	0.065
19. Is picked on or bullied by other youth		−0.532	0.057
11. Has at least one good friend		0.539	0.055
18. Often lies or cheats	Conduct problems	0.551	0.051
22. Steals from home, school or elsewhere		0.312	0.062
5. Often loses temper		0.581	0.050
12. Often fights with other youth or bullies them		0.395	0.058
7. Generally well-behaved, usually does what adults request		−0.647	0.046

### Reliability analyses

To assess the reliability of the SDQ subscales, Cronbach’s alpha was computed. The Cronbach’s α internal consistency coefficient (see Table 2) was good for the SDQ Total Difficulties Scale (α = 0.750), the hyperactivity-inattention subscale (α = 0.813), the emotional symptoms subscale (α = 0.764) and the prosocial behaviors subscale (α = 0.740), as their Cronbach’s alpha exceeded the recommended 0.7 threshold values (Cohen, 1977). While lower, the peer problems (α = 0.677) and the conduct problems (α = 0.670) subscales were still deemed acceptable.

## Discussion

### Summary of results

The present study examined the factor structure of the parent-rated version of the SDQ based on a sample of Canadian children from military families. The SDQ is a 25-item questionnaire that is frequently used to assess children’s psychosocial attributes, including both positive and negative behaviors, and is comprised of five subscales: Emotional Symptoms, Hyperactivity-Inattention, Peer Problems, Conduct Problems and Prosocial Behavior. This study used exploratory factor analysis to determine the factor structure for this particular population and examine whether the findings from this study supported earlier research regarding the psychometric properties of the instrument. While a sixth factor did emerge in the initial model, it was found to contribute to only 2% of the total variance with only two items loaded. As such, a second exploratory factor analysis was performed based on a fixed five-factor model. Confirmatory factor analysis was next conducted to validate the factor structure identified through the exploratory factor analysis and compare the results to other factor structures that have been presented in the literature. This study provides evidence for the overall soundness of the five-factor structure as originally proposed (Goodman, 1997), with results providing support for the model for the parent-rated SDQ using Canadian military children aged 3–16 years. However, good fit was observed for some indices but not all which suggests there may be ways to improve the model. For example, removing items that may be problematic such as those with lower factor loadings, those which cross-loadings or those that do not load as expected according to the proposed framework may help to improve model fit.

### Comparison to previous research

The findings of this study are largely consistent with other research on the SDQ conducted on children from civilian families across a range of global and cultural contexts (e.g., Smedje et al., 1999; Hawes and Dadds, 2004; Van Roy et al., 2008; Goodman et al., 2010; He et al., 2013; Niclasen et al., 2013; Kersten et al., 2016), including children within a Canadian context, in which studies demonstrated the robustness of the measure across different subsamples of the youth population in Canada and confirmed the five-factor structure (Aitken et al., 2015; Hoffmann et al., 2020).

The Conduct Problems item, “*Generally well-behaved, usually does what adults request*” cross-loaded into the Prosocial scale. Hawes and Dadds (2004) note that, while this may appear to be conceptually confusing, it is not inconsistent with past research which has also reported unexpected factor loadings for this “obedience” item (e.g., Thabet et al., 2000; Goodman, 2001; Muris et al., 2003; Dickey and Blumberg, 2004). They note that such findings have contributed to the prompting of the Prosocial scale in being described as a “positive” factor and the results from this study similarly further add to the existing evidence that questions the value of categorizing this item as an indicator of conduct problems in children. Within the Emotional Symptoms subscale, the item, *“Is often unhappy, depressed or tearful,”* cross-loaded onto Peer relationship Problems which did not correspond to what the literature would suggest. It is possible that, collectively, such findings are reflective of some factors being more unitary than others, with a small number of items cross-loading or loading weakly on their subscales. Regardless, the pattern of fit and significant factor loadings overall confirmed the five subscales and model structure found in past research (Aitken et al., 2015).

Internal reliability of the SDQ was also supported in this study, with Cronbach’s alpha coefficients above 0.70 for the Total Difficulties score and deemed good for three subscales (i.e., Hyperactivity-Inattention, Emotional Symptoms and Prosocial Behavior), Similar to other studies, lower reliability coefficients were observed for the Conduct and Peer Problems subscales (Smedje et al., 1999; Goodman, 2001; Koskelainen et al., 2001; Muris et al., 2003; Mansbach-Kleinfeld et al., 2010), although they still met an acceptable threshold. Speculation as to the low internal consistency values of these subscales has attributed it to the possibility that it measures more heterogeneous constructs than intended or that it may be due to several positively worded reverse-scored items included in these subscales (Smedje et al., 1999; Muris et al., 2004; Palmieri and Smith, 2007; Mansbach-Kleinfeld et al., 2010).

### Limitations

This study used data collected from spouses and partners of CAF members who were asked to complete a self-administered questionnaire assessing their quality of life that included the SDQ intended for respondents with children aged 3–16 years. There are several potential limitations of this study that should be acknowledged. First, the relatively low response rate of the survey limits the ability to generalize the results of this study to the greater population of Canadian children from military families, as non-respondents may differ from respondents in ways that influence reporting (Hill and Hughes, 2007). It is possible that those who responded were more likely to have a poorer quality of life and/or for their children to be experiencing greater emotional or behavioral challenges. As such, the extent to which the present sample is representative of the total population is unknown. Also, the lack of additional measures of child behavior and functioning that were included in the QoL survey limited the ability to validate the SDQ data.

Additionally, this data is based on a single parent assessment with no other informants reporting on the children included in this study. It is therefore difficult to discern the degree to which there may have been either an under- or over-reporting of problematic behaviors (Goodman et al., 2004; Mansbach-Kleinfeld et al., 2010). Gathering data from multiple informants who observe the children across different contexts or who may have separate perspectives on the functioning of the children would have enabled for an evaluation of mental health and behavioral problems that is more comprehensive, as well as accurate (Palmieri and Smith, 2007; Kersten et al., 2016; Español-Martín et al., 2021). Future research including additional informants would help reduce the potential for bias captured in the data and enable for cross-validation of reports for this population of Canadian military children.

While it was beyond the scope of this study to conduct a meaningful and valid analysis of gender differences in the sample, it is possible that the gender of the child being reported upon influenced the results (Mellor, 2005; Mansbach-Kleinfeld et al., 2010; Niclasen et al., 2013; Español-Martín et al., 2021). As well, the sample used in the study included all children from the ages of 3 to 16 years, with the majority between the ages of 3 to 8 years old. There is potential that such a broad age span in this sample masked potential differences between subgroups (Niclasen et al., 2013).

Although children from military families may be considered to be at higher risk for displaying emotional and behavioural challenges due to the demands and stressors of military life (Chandra et al., 2010; Blamey et al., 2019; Cramm et al., 2019; Mahar et al., 2022), the children of the respondents in this study were largely found to score within the “normal” range for all five subscales and in the total scoring of the SDQ, with smaller proportions found to fall within the “borderline” or “abnormal” categories. The respondents of this study also were drawn from a random stratified sample of all military spouses across Canada, capturing data on the general population as whole, and did not specifically target those with children identified as displaying problematic behaviour and/or emotional symptoms. In this sense, the children in this study would arguably be considered part of a low-risk population as compared to children with a diagnosed mental health condition or those who would meet clinical definitions of high-risk, such as children receiving treatment or who were in care. It may therefore be a limitation that the analysis was based data collected from a low prevalence population, with relatively small rates of any kind of maladaptive or problematic behavior observed in the sample (Goodman et al., 2010; Niclasen et al., 2013). However, the instrument is still considered appropriate for screening low-risk children from a population in which the majority of children are healthy (Palmieri and Smith, 2007), which is reflective of the sample used in the study based on the scoring assessment of the SDQ results.

Lastly, the influence of the cultural and social differences of a Canadian population of military families on the factor structure of the SDQ remains unclear. Although there has been a global and widespread use of the SDQ (e.g., He et al., 2013; Niclasen et al., 2013; Español-Martín et al., 2021), it has not been fully normed on a Canadian sample, thereby necessitating a reliance on normative information based primarily from work conducted in the United States and the United Kingdom (Aitken et al., 2015). Similarly, the present study also included only the original British version of the SDQ with no modifications made to adapt it to either a Canadian or a military-specific environment. The French questionnaire was based on a professional translation of the original English version and respondents were given the opportunity to complete the survey in the official language of their choice. Approximately one-quarter of respondents completed the survey in French and/or reported that French was their first official language. Although not necessarily definitive for this sample, Hoffmann et al. (2020) in their study of the SDQ based on a nationally representative sample of Canada children did find that configural, metric and scalar invariance was supported for survey language (English vs. French). However, it is possible that that there may be some linguistic or cultural nuances that were not sufficiently captured. Given that Canada is a diverse and multicultural country with two official languages and that there may be a further distinct contextual influence amongst military families, interpretations of the results may not be generalizable to all Canadian Armed Forces children across the country.

## Conclusion

To our knowledge, this is the first study that has examined the factor structure of the SDQ within the context of Canadian military children. Despite the potential limitations of this tool, this study demonstrates the value in using this instrument to assess the emotional and behavioral well-being of children from military families in Canada, particularly if the factor structure is replicated in future research. Results of this study provide evidence for the factorial validity of the parent-rated SDQ with a sample of children from military families. Specifically, it supports the original five-factor solution and aligns with past research findings on the SDQ. Internal reliabilities of the total scale as well as the subscales were found to meet an acceptable threshold. While it was beyond the scope of this study, future research should examine differences by demographic subgroup of the children, such as age, gender and first official language, as well as utilize multiple informants.

## Data availability statement

The datasets presented in this article are not readily available because due to the nature of this research, participants of this study did not agree for their data to be shared publicly, so supporting data is not available. Requests to access the datasets should be directed to julie.coulthard@forces.gc.ca.

## Ethics statement

The studies involving human participants were reviewed and approved by Department of National Defence Social Science Research Review Board (SSRRB Approval #1650/17f). The patients/participants provided their written informed consent to participate in this study.

## Author contributions

JC and KS conceptualized and designed the study. JC and KS conducted separate parts of the analysis and shared all results interpretation. JC wrote the initial version of the manuscript. KS contributed to the revisions as well as conducted general editing. All authors contributed to the article and approved the submitted version.

## Conflict of interest

The authors declare that the research was conducted in the absence of any commercial or financial relationships that could be construed as a potential conflict of interest.

## Publisher’s note

All claims expressed in this article are solely those of the authors and do not necessarily represent those of their affiliated organizations, or those of the publisher, the editors and the reviewers. Any product that may be evaluated in this article, or claim that may be made by its manufacturer, is not guaranteed or endorsed by the publisher.

## Footnotes

^1^ http://www.sdqinfo.com
